# Effects of Cryotherapy on the Maxillary Antrostomy Patency in a Rabbit Model of Chronic Rhinosinusitis

**DOI:** 10.1155/2013/101534

**Published:** 2013-10-28

**Authors:** Anamaria Gocea, Marian Taulescu, Veronica Trombitas, Silviu Albu

**Affiliations:** ^1^II-nd Department of Otolaryngology, Iuliu Hatieganu University of Medicine and Pharmacy Cluj-Napoca, Street Republicii No. 18, 400015 Cluj-Napoca, Romania; ^2^Pathology Department, University of Agricultural Sciences and Veterinary Medicine, 400372 Cluj-Napoca, Romania

## Abstract

It is acknowledged that many causes of failures in endoscopic sinus surgery are related to scarring and narrowing of the maxillary antrostomy. We assessed the effect of low-pressure spray cryotherapy in preventing the maxillary antrostomy stenosis in a chronic rhinosinusitis (CRS) rabbit model. A controlled, randomized, double-blind study was conducted on 22 New Zealand rabbits. After inducing unilateral rhinogenic CRS, a maxillary antrostomy was performed and spray cryotherapy was employed on randomly selected 12 rabbits, while saline solution was applied to the control group (*n* = 10). The antrostomy dimensions and the histological scores were assessed 4 weeks postoperatively. The diameter of cryotreated antrostomy was significantly larger at 4 weeks than that in the control group. At 4 weeks, the maxillary antrostomy area in the study group was significantly larger than the mean area in the control group (103.92 ± 30.39 mm^2^ versus 61.62 ± 28.35 mm^2^, *P* = 0.002). Submucosal fibrous tissues and leukocytic infiltration in saline-treated ostia were more prominent than those in cryotreated ostia with no significant differences between the two groups regarding the histological scores. Intraoperative low-pressure spray cryotherapy increases the patency of the maxillary antrostomy at 4 weeks postoperatively with no important local side effects.

## 1. Introduction

Endoscopic sinus surgery (ESS) is highly effective in curing medically resistant maxillary chronic rhinosinusitis (CRS). Nevertheless, between 2 and 18% of cases require revision surgery [1–3] due to the following failure reasons: middle turbinate lateralization, mucosal adhesions, vicious scarring, and ostium narrowing. It is accounted that up to 25% of maxillary ESS failures are related to ostium stenosis [1–4]. In order to enhance success rates, various methods were forecasted: mucosal sparing surgical techniques [5], postoperative endoscopic debridements [6], perioperative drug-infused dressings [7–13], bioabsorbable drug-coated stents [14–18], mucoadhesive drug-eluting polymers [19, 20], and the use of oral and topical corticosteroids [21].

Cryotherapy, as a surgical tool, has been extensively used until nowadays in oncology, ophthalmology, and gastroenterology [22, 23]. Unlike the cryoprobe (−86°C), spray cryotherapy with liquid nitrogen (−196°C) is a noncontact method of tissue ablation that can be used to quickly treat larger areas, providing more uniform treatment. A study in the airway of humans [24] was conducted using surgically resected specimens that determined cryotherapy's safety and feasibility. The recent reports of successful noncontact low-pressure spray cryotherapy [25, 26] to modify the wound response in granulation-induced glottic and subglottic stenosis have prompted us to investigate its effect on mucosal healing after ESS.

Our objective was to study the outcomes of the use of low-pressure spray cryotherapy on the surgically created maxillary antrostomy in an experimental CRS rabbit model. Our hypothesis was that cryotherapy is able to reduce stenosis of the antrostomy during the postoperative period. Since ESS is the treatment of choice in CRS cases, we chose to examine the effect of cryotherapy on long-term inflamed mucosa.

## 2. Materials and Methods

This study was approved by the University Committee on Animal Care & Use and the animals were treated according to the National Institute of Health Guide for the Care & Use of Laboratory Animals. We designed a prospective, controlled, randomized, double-blind, and parallel-group animal study on a total of 24 New Zealand white rabbits of both genders with body weights ranging from 2.5 to 3.2 kg.

### 2.1. Animal Model and Surgical Technique

Initially, unilateral rhinogenic CRS according to the method described by Liang et al. [27] was induced. Briefly, rabbits were anesthetized with ketamine (50 mg/kg i.m.) and xylazine (4 mg/kg i.m.). Under endoscopic control, 1 *μ*g phorbol 12-myristate 13-acetate (PMA, Sigma-Aldrich, St. Louis, MO, USA) was injected into unilateral nasal lateral wall near the endoturbinates (similar to middle turbinates in humans) [16]. The specific sides to be injected were randomly generated by a computer program.

Afterward, a 3 × 5 × 30 mm piece of Merocel (Medtronic Xomed) was inserted into the nasal cavity. The Merocel was big enough to ensure ostial occlusion and it was removed 15 days later. As demonstrated by Liang et al., this model is able to induce persistent inflammation lasting for more than 12 weeks, meeting the current definition of CRS [27].

At the end of the three-month period, we performed a maxillary antrostomy on the infected sinus. The operative technique was standardized and performed as described in the literature [20, 27–29]. After induction of anesthesia as previously described, a T-shaped incision was made over the lateral nasal dorsum through the skin and periosteum. Both maxillary sinuses were inspected. The superior wall of the infected sinus was completely opened rendering the natural ostium visible. The ostium was circumferentially widened by a cutting bur, creating a through-and-through wound; we sent the mucosa samples removed around the maxillary ostium for histological examination and we took digital photographs of the antrostomies.

Before wounding, rabbits were randomly assigned to one of the two treatment groups. In Group 1 (12 rabbits), cryotherapy was employed (two cycles of 4-second cryospray with complete thaw of the treated area between applications), while animals within Group 2 (12 rabbits) were sprayed with saline solution at the same dosage and period. Spray cryotherapy was performed with the CryoPro Cryotherapy System (Williams Medical Supplies Ltd), a device that provides a uniform and broad distribution of liquid nitrogen −196°. Precise handling of the device allowed spraying the liquid nitrogen for 4 seconds on the maxillary antrostomy circumference, and then we waited 35 seconds for thawing the sprayed area; afterwards, we applied the last cycle 4 seconds. We carefully avoided overspraying the liquid nitrogen on the maxillary sinus mucosa or the nasal fossa (through the ostium) in order to prevent any interference with the study results and not to damage surrounding tissues. The periosteum, subcutaneous tissue, and skin were closed with 4–0 Vicryl suture. Rabbits received antibiotics (30 mg/kg i.m ceftriaxone) for 10 days and were closely monitored.

### 2.2. Antrostomy Dimensions

**Figure 1:**
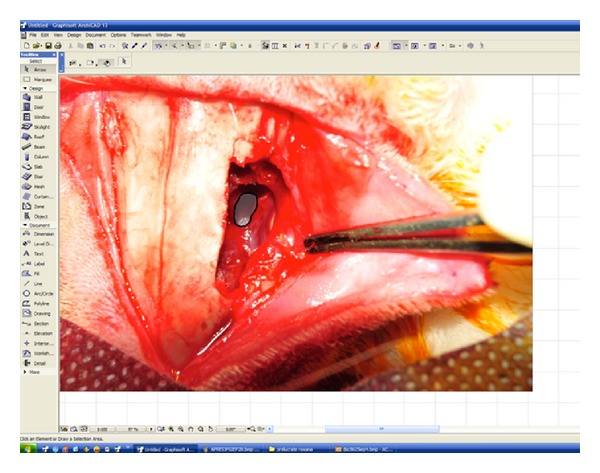
Measuring the antrostomy area.

At the end of the 4th postoperative week, the rabbits were sacrificed by intravenous administration of 500 mg phenobarbital. Immediately after their death, we reopened the midline incision to permit access to the maxillary sinus. A blinded observer inspected the sinuses and extracted mucosa samples for histological analysis; local aspects were documented by digital photography. The pictures were taken by a professional photographer from the same camera angle respecting the same focal distance, minimalising as much as possible the bias of different angled pictures for the digital area measurement. All pictures were loaded on the Graphisoft ArchiCAD 13 program and the antrostomy area was objectively measured in an automated manner (Figure 1). The patency of an ostium was scored by comparing its area value at 4 weeks with its area value at the date of the surgical procedure.

### 2.3. Histological Analysis

Mucosa specimens obtained around each ostium were immediately fixed in 10% phosphate-buffered formalin solution for 24 hours, embedded in paraffin wax, cut into 5–7 *μ*m sections, and stained with hematoxylin and eosin (H&E). Masson's trichrome (M&T) staining was also done for evaluating collagen fibers. The slides were analyzed with an Olympus BX51 microscope with an Olympus SP 350 digital camera.

“Cell B” basic imaging software (Olympus) was used for semiautomatic counting of the inflammatory parameters. Morphological evidence of epithelial damage such as cilia disappearance, disruption of epithelium or inflammatory cell infiltrates, fibrosis, and edema was looked for under light microscopy and assessed according to the scale represented in Table 1. The pathologist was blinded, unaware if the samples were harvested at the time of surgery or 4 weeks later or if they came from rabbits in the control or the study group.

### 2.4. Statistical Methods

The statistical analysis was performed by means of SPSS 20.0 (SPSS, Inc., Chicago, IL, USA). Data were expressed as mean ± standard deviation (SD). *P* values < 0.05 were considered significant. We used the Student's *t*-test for normally distributed data, while the nonparametric test Mann-Whitney *U* test was applied for numbers that did not follow a normal distribution. Wilcoxon rank test assessed differences in treatment outcomes because the number of animals was limited and the data were not normally distributed. Correlation analysis was performed using Spearman statistics.

## 3. Results

Twenty-two rabbits (12 animals in Group 1 and 10 rabbits in Group 2) survived until the end of the study; one animal died because of an anesthetic accident and another one died during the 3-month period of chronic sinusitis induction time; the latter ate very poorly for a few days probably due to a dental abscess, rather frequent in rabbits. Unilateral mucopurulent nasal discharges were observed in all 22 rabbits at the end of the 3-month maxillary sinusitis induction time. At surgery, all the ostium-occluded maxillary sinuses were filled with purulent secretions and hypertrophic mucosa. However, the contralateral sinus was free of disease in all animals.

### 3.1. Chronic Rhinosinusitis Model

**Figure 2:**
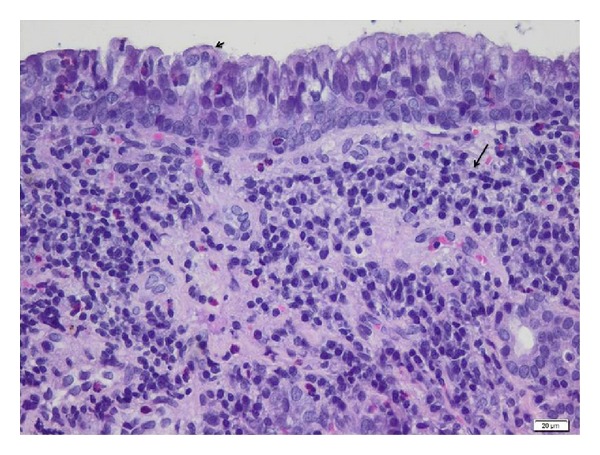
Mucosa around the ostia at the moment of surgical intervention-light microscopy. Maxillary sinus biopsy showing thickening of sinus mucosa, lymphoid follicle hyperplasia, cilia degeneration (black arrow head), diffuse and several infiltrate with mononuclear cells (black arrow), heterophils, and scattered eosinophils. H&E stain, bar = 20 *μ*m.

Microscopic analysis of mucosal specimens collected at surgery demonstrated thickening of sinus mucosa, epithelial hyperplasia, mucous metaplasia, moderate to severe subepithelial fibrosis, and glandular atrophy. A prominent leukocytic infiltration into the lamina propria and epithelium was observed, with predominant mononuclear cell infiltrates (lymphocytes, macrophages, and plasma cells) and lymphoid follicle hyperplasia (see Figure 2). A moderate infiltrate with heterophils and eosinophils was also observed. No nasal polyps were found either macroscopically or microscopically in this study. Discrete inflammation (between 10 and 30 monocytes/field 40x) was found in 2 rabbits (9.09%) and grade 2 (moderate inflammation) was found in 31.81% (7 rabbits), and the majority (59.1%—13 rabbits) presented with grade 3 of severe inflammation (>50 mononuclear cell infiltrate/field 40x) at the time of surgery.

### 3.2. Antrostomy Dimension

**Figure 3:**
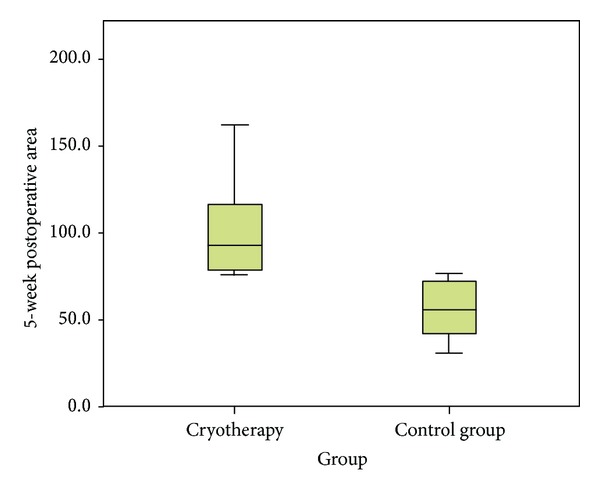
Antrostomy area distribution in the two groups.

All wounds healed without infection and all antrostomies remained open in 4 weeks' time. In the fourth postoperative week, there were no cryotherapy-induced changes to the periantrostomy sinus mucosa, so we consider that the blinding was accurate.  Table 2 shows the mean diameter and area values of the maxillary ostia in both animal groups. Initial dimensions and diameters of the antrostomies were identical in both groups. At 4 weeks, the Group 1 ostia were statistically significantly wider than saline-treated ostia (see Figure 3). At 4 weeks, the antrostomy patency in study group has enlarged by a mean of 47.53%, while in the control group, it has narrowed by 20.06% (statistically significant difference, *P* < 0.05). Cryotherapy was able to significantly enlarge the antrostomy as compared with that of the control group (*P* < 0.05 for both area and diameter, Wilcoxon sum rank test). A direct correlation was found between the histology scores (mean epithelial height, mononuclear cell infiltrate, fibrosis, and edema) and the mean ostial diameter for each of the two treatments (*r* = 0.548, *P* < 0.05, Spearman *r* test for correlation).

### 3.3. Histological Analysis

**Figure 4:**
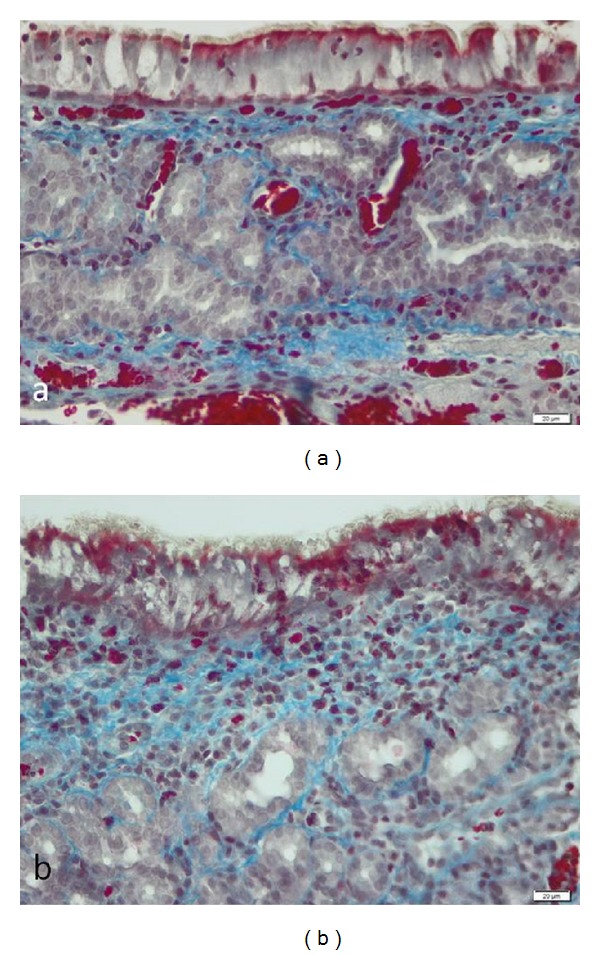
The light microscopic findings of Masson's trichrome (M&T) stained mucosa around the ostia at 4 weeks in cryotreated mucosa: subepithelial collagen fiber depositions stained with blue color (a), bar = 20 *μ*m, were less prominent compared with saline-treated mucosa (b), bar = 20 *μ*m.

**Figure 5:**
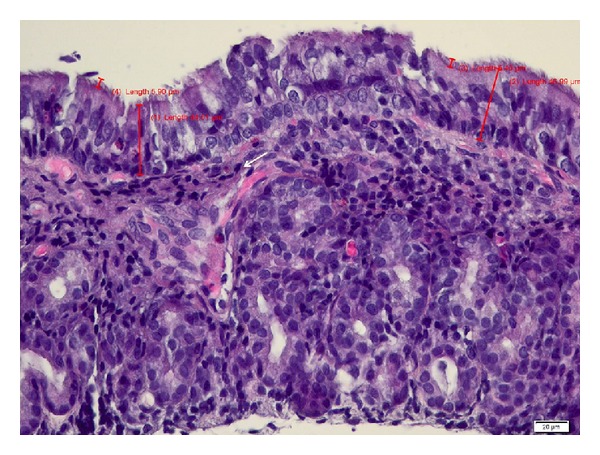
The histological exam of mucosa around the ostia at 4 weeks in the cryotreated group revealing epithelial hyperplasia, scattered leukocytes, and moderate subepithelial fibrosis (white arrow). H&E stain, bar = 20 *μ*m.

The histology scores at the end of the study are displayed in Table 3. Postoperative 4-week specimens stained with Masson's trichrome showed that even though fibrosis score is higher in saline-treated ostia as compared to that in cryotreated ostia (Figure 4), the difference did not attain statistical significance (see Table 3). The mean value of the epithelial height decreases at 4 weeks to 36.02 ± 12.04 *μ*m even though it does not reach the normal values. In the cryotreated group, it decreased in a statistically significant way (*P* < 0.05) from 51 *μ*m to 33 *μ*m, while in the control group the mean height of epithelium suffers only a slight variation (3 *μ*m). There was an increase in edema in both groups, but the difference was not statistically significant (see Table 3). Fibrosis decreases in the study group after 4 weeks, from a mean value of 2.25 to 1.85, the difference not being statistically significant (*P* > 0.05; see Table 3). We found a better organization of collagen fibers in the study group versus the control group. There was no significant difference between the control and study groups with respect to loss of cilia. More areas with normally ciliated epithelia were found in the cryotreated group even though their function has not been assessed. Cellular atypia was not observed in any of the samples. Light microscopic findings of the mucosal specimens obtained from the cryotreated group showed epithelial hyperplasia (Figure 5) after 4 weeks in four cases (33.3%), all the rest presenting a normal architecture of the epithelial layer with some inflammatory cells in the submucosal planes.

## 4. Discussion

Our histopathological results showed severe and moderate degrees of inflammation in 20 rabbits at the time of surgery, reinforcing the study's clinical significance: endoscopic sinus surgery is indicated for chronic severe disease persisting for months, sometimes years, in spite of repeated appropriate antibiotic and steroid therapy. The sinus mucosa in the rhinosinusitis-induced rabbits was characterized by eosinophil-dominant inflammation, similar to the results reported by Johnston et al. [23] Thus, we succeeded in reproducing an important experimental model of chronic rhinosinusitis without nasal polyps. Maintaining the patency of maxillary antrostomy is one of the goals of successful endoscopic sinus surgery. Four weeks after the surgical intervention, the cryotreated maxillary antrostomies were significantly wider than the saline-treated ostia.

The anteroinferior end of the middle turbinate and the lateral nasal wall delineate an isthmus, which is prone to synechia formation and ostium stenosis [1–3] during the stage of postoperative edema. Endoscopic debridements were recommended in order to prevent adhesions following ESS. This painful procedure enables observation and management of scarring but may also slow down wound healing [19]. Therefore, as mentioned above, different devices were foreseen to promote middle meatus and ostium patency. In this study, we assumed that cryotherapy may hinder ostium stenosis.

Experimental animal models are good tools for understanding the pathogenesis and envisaging treatment in CRS [27–30]. Likewise, the maxillary sinus of rabbits is considered to be a good experimental model for regenerative studies following sinus surgery [27–33]. Several models [27–34] of sinusitis induction have been described in the literature; the majority of them are for acute disease. We chose Liang's method because it is simple to perform and feasible, and it avoids the need of manipulating pathogenic bacteria. Since ESS is the method of choice in caring for CRS, we thought this model would best replicate the clinical setting of performing middle meatal antrostomy during surgery. In other experimental models, the mean diameter of antrostomy was 4 mm [19, 20, 29]. We choose the 8 mm diameter since, as opposed to the previous models in the literature, we operated on infected sinuses, and in this setting a wide antrostomy is recommended by most authors [5].

The mechanism of action of cryotherapy includes disruption of cell membranes by the formation of intracellular ice crystals during the freeze cycle and vasoconstriction, endothelial damage, thrombosis, and tissue ischemia during the thaw cycle [22, 23]. Basic and clinical research demonstrated that cryotherapy induced the production of collagen type III along with the development of more organized collagen architecture [24]. Wound healing is a significant determinant of successful outcomes in endoscopic sinus surgery. In the human airways, histological evaluations showed a better wound healing following cryotherapy including improved collagen organization and decreased keratinization [24, 25]. Moreover, the supporting connective matrix was left intact after cryotherapy application and long-term pathology findings revealed a complete lack of scarring or stricture [26]. Therefore, patients who exhibit persistent mucosal disease after ESS, which leads to repetitive antibiotic treatment and even surgical revisions, might benefit from intraoperative cryotherapy application, since it modulates mucosal healing and decreases granulation tissue formation.

Our study demonstrated that cryotherapy significantly improved the antrostomy patency. At 4 weeks, the ostium diameter was significantly bigger in the cryotreated group. Further, the mean area in the cryotreated group has increased by 47.53% (including by default the cryoablation effect), while in the control group it has narrowed by 20.06% following the normal process of mucosal healing by granulating tissue. No mucosal toxic effects were observed in Group 1. On the contrary, histology demonstrated that submucosal fibrous tissues and leukocytic infiltration in the control group ostia were more prominent than those in cryotreated ostia. In addition, a better organization of collagen fibers and more areas with normally ciliated epithelia were found in the cryotherapy group. Improved ciliated epithelia maintain normal drainage of sinuses which is based on mucociliary transport function regardless of maxillary antrostomy patency.

There are several limitations to our study. Firstly, we did not split the study rabbits into several groups to which we should have applied different amounts of cryotherapy for different cycles in order to find out the most appropriate dosage. Although cilia appeared to be intact in the cryotreated group, their function was not appropriately assessed. Moreover, electronic microscopy analysis of the treated cilia and measurement of cellular markers for inflammation and wound repair might have made our conclusions about the use of cryotherapy more accurate. Another flaw is that we should have measured the antrostomy diameter after application of cryotherapy and should have assessed it at different time intervals during the study. Previous work of Proctor et al. [29] with the noninfected rabbit model of ostium patency used a 3-week end point for analysis. On the other hand, Chen et al. [20] assessed the neo-ostium diameter at 2, 3, and 4 weeks postoperatively in their study on the hyaluronan hydrogels dressing in the rabbit maxillary sinus. In the latter study, control at 3 weeks simulated postoperative debridement in human ESS [20]. Since we assume that cryotherapy does not imply debridement, we set the 4-week end point for our investigation. Another major flaw is the restricted observation period of our study. However, we were guided by the abovementioned papers on experimental sinusitis in rabbits, choosing a similar observation period [27–34]. Moreover, it is very well known that small animals heal more quickly than humans, and this ability to maintain a patent antrostomy for at least 4 weeks would equate to a useful clinical effect on humans for about 10 months (since one month in a rabbit's life stands for about 10 months in a human's [31–34]). An important limitation of our study is also the absence of specific histological examination (Alcian blue staining to detect hyaluronic acid, Movat's staining to detect elastin fibers, and picrosirius-polarization staining to detect collagen fibers [25]) performed at defined time intervals—1, 2, 3, 4, 6, 8, and 12 weeks. Because of this drawback, we were not able to reveal the mechanism underlying the enlarged antrostomy in the cryotherapy group. Based on the experimental data in the larynx [25], we can only suppose that in an inflammatory sinus setting, cryotherapy induces local necrosis and the wound healing is characterized by a better organized connective tissue structure.

## 5. Conclusion

In conclusion, intraoperative low-pressure spray cryotherapy increases the patency of the maxillary antrostomy at 4 weeks postoperatively with no important local side effects. Further studies are needed in order to determine the best dosage for effects of cryotherapy. Moreover, in order to demonstrate the effectiveness and the safety of cryotherapy in maxillary antrostomy, longer-term observation and careful histological examination might be needed. Based on solid experimental evidence, further clinical trials of the use of spray cryotherapy in ESS might be attempted, such as enhancing the middle meatus antrostomy and frontal recess patency rates and decreasing dacryocystorhinostomy failure rates.

## Figures and Tables

**Table 1:** Grading of histological parameters evaluated during the study.

Parameter	Grade	Description
Mononuclear cell infiltrate	Grade 0	Normal aspect (between 0 and 10 cells/field 40x)
	Grade 1	Discrete inflammation (between 10 and 30 cells/field 40x)
	Grade 2	Moderate inflammation (between 30 and 50 cells/field 40x)
	Grade 3	Severe inflammation (>50 cells/field 40x)

Fibrosis	Grade 0	Normal (between 3 and 4 subepithelial layers of collagen fibers)
	Grade 1	Subepithelial fibrosis
	Grade 2	Subepithelial and interglandular fibrosis
	Grade 3	Diffuse fibrosis with compression atrophy of adjacent structures (glands and capillaries)

Edema	Grade 0	No edema
	Grade 1	Focal subepithelial edema with collagen fibers dislocation
	Grade 2	Diffuse subepithelial edema with collagen fibers dislocation
	Grade 3	Diffuse edema (subepithelial and interglandular)

Cilia	Grade 0	Normal aspect (height: 4.5–5 *µ*m)
	Grade 1	Shortened cilia
	Grade 2	Dotted cilia disappearance (epithelial areas without cilia)
	Grade 3	Lack of cilia on the epithelium of the inflammatory areas

Epithelial hyperplasia	Grade 0	Absent
	Grade 1	Present

**Table 2:** Mean values of antrostomy diameters and areas for both animal groups.

	Group 1 (*N* = 12) mean ± SD	Group 2 (*N* = 10) mean ± SD	Statistics	*P* value
Initial diameter (mm)	8.14 ± 2.31	8 ± 1.82	Mann-Whitney *U* test	0.005
Final diameter (mm)	9.73 ± 2.92	7.2 ± 2.61		

Initial area (mm^2^)	72.86 ± 23.62	74.79 ± 25.43	Mann-Whitney *U* test	0.002
Final area (mm^2^)	103.92 ± 30.39	61.62 ± 28.35		

Group 1: cryotherapy.

Group 2: control.

SD: standard deviation.

**Table 3:** Histology scores.

Parameter	Group 1 *N* = 12 mean ± SD	Group 2 *N* = 10 mean ± SD	Statistics	*P* value
Mean epithelial height	38.02 ± 12.96	33.62 ± 11.52	*T*-independent test	0.415 (95% confidence interval: −6.62437–15.41171)
Edema	1.83 ± 1.267	1.80 ± 1.033	Chi square	0.505 (Chi Sq Val = 2.338)
Fibrosis	1.75 ± 1.422	1.60 ± 1.174	Chi square	0.417 (Chi Sq Val = 2.842)
Cilia	1.36 ± 1.206	1.00 ± 1.054	Chi square	0.774 (Chi Sq Val = 1.113)
Mononuclear cell infiltrate	1.42 ± 0.996	1.40 ± 1.075	Mann-Whitney	0.945 (*U* = 59)

Group 1: cryotherapy.

Group 2: control.

SD: standard deviation.
